# Topical Anti‐Inflammatory and Analgesic Effects of Multiple Applications of S(+)‐Flurbiprofen Plaster (SFPP) in a Rat Adjuvant‐Induced Arthritis Model

**DOI:** 10.1002/ddr.21314

**Published:** 2016-05-31

**Authors:** Masanori Sugimoto, Yoshihisa Toda, Miyuki Hori, Akiko Mitani, Takahiro Ichihara, Shingo Sekine, Shinsuke Kaku, Noboru Otsuka, Hideo Matsumoto

**Affiliations:** ^1^ Research Headquarters, Taisho Pharmaceutical Co., Ltd.SaitamaJapan; ^2^ Development Headquarters, Taisho Pharmaceutical Co., Ltd.TokyoJapan; ^3^ Institute for Integrated Sports Medicine, Keio University School of MedicineTokyoJapan

**Keywords:** S(+)‐flurbiprofen, analgesic, anti‐inflammatory, NSAIDs

## Abstract

**Table:** 

Preclinical Research

The aim of this study was to evaluate the efficacy of multiple applications of S(+)‐flurbiprofen plaster (SFPP), a novel Nonsteroidal anti‐inflammatory drug (NSAID) patch, for the alleviation of inflammatory pain and edema in rat adjuvant‐induced arthritis (AIA) model as compared to other NSAID patches. The AIA model was induced by the injection of *Mycobacterium butyricum* and rats were treated with a patch (1.0 cm × 0.88 cm) containing each NSAID (SFP, ketoprofen, loxoprofen, diclofenac, felbinac, flurbiprofen, or indomethacin) applied to the paw for 6 h per day for 5 days. The pain threshold was evaluated using a flexion test of the ankle joint, and the inflamed paw edema was evaluated using a plethysmometer. cyclooxygenase (COX)−1 and COX‐2 inhibition was evaluated using human recombinant proteins. Multiple applications of SFPP exerted a significant analgesic effect from the first day of application as compared to the other NSAID patches. In terms of paw edema, SFPP decreased edema from the second day after application, Multiple applications of SFPP were superior to those of other NSAID patches, in terms of the analgesic effect with multiple applications. These results suggest that SFPP may be a beneficial patch for providing analgesic and anti‐inflammatory effects clinically. Drug Dev Res 77 : 206–211, 2016. © 2016 The Authors Drug Development Research Published by Wiley Periodicals, Inc.

## Introduction

Nonsteroidal anti‐inflammatory drugs (NSAIDs) exert their pharmacologic and toxicological effects mainly through the specific inhibition of arachidonic acid binding to cyclooxygenase (COX), inhibiting the production of prostaglandins (PGs) including PGE_2_, a major imediator of inflammation, pain, and fever [Narumiya et al., 1999]. NSAIDs are widely used to relieve the chronic pain associated with musculoskeletal disorders, such as rheumatoid arthritis (RA) and osteoarthritis (OA), and are available as both oral and topical formulations for clinical application. Although the efficacy of oral NSAIDs is well established, their use is known to have a risk factor for serious gastrointestinal (GI) disturbances [Wolfe et al., 1999; Huang et al., 2002; Sakamoto et al., 2006]. Conversely, the topical administration of NSAIDs is associated with a lower risk of adverse GI effects [Heyneman et al., 2000]. A drawback in the use of topical NSAIDs is their low absorption rate [Taburet et al., 1995]. Thus, a topical patch containing a potent NSAID that is absorbed more efficiently would potentially be an excellent solution for relieving chronic pain without severe adverse effects.

We developed a novel topical NSAID patch, the S‐flurbiprofen plaster (SFPP), containing the (S)‐enantiomer of flurbiprofen (SFP) which is more potent as COX inhibitor than R(‐)‐flurbiprofen. COX inhibition produced by flurbiprofen is predominantly attributable to SFP [Peskar et al., 1991; Carabaza et al., 1996]. We previously showed that SFP potently inhibits human COX‐1 and COX‐2, compared with ketoprofen and loxoprofen. Moreover, SFPP exhibited the highest absorbability, compared with ketoprofen and loxoprofen patches in rats [Sugimoto et al., 2016]. Transdermal absorption of SFPP is improved by the addition of certain excipients [Yataba et al., 2016]. Based on these preferable characteristics, we examined the potent inhibitory effect of a single application of SFPP on PGE_2_ levels in paw exudate and inflammatory pain in a rat adjuvant‐induced arthritis (AIA) model as compared with ketoprofen and loxoprofen patches. SFPP rapidly inhibited PGE_2_ and inflammatory pain in the rat AIA model [Sugimoto et al., 2016]. However, the application of only a single patch was evaluated in this study, and the therapeutic efficacy of repeated applications of SFPP, simulating a clinical regimen, remains to be elucidated for the prediction of a possibly beneficial clinical effect. In addition, the anti‐inflammatory effect of SFPP also has not yet been examined in a rat AIA model.

In this study, we compared the human COX inhibition activity of SFP with active ingredients from other major NSAIDs patch. We also compared the analgesic and anti‐inflammatory effects after repeated applications of SFPP and a series of clinically available NSAID patches in the rat AIA model.

## Materials and Methods

### Drugs

The following products were evaluated in this study: SFPP (40 mg/140 cm^2^, Tokuhon Corporation, Tokyo, Japan), ketoprofen patch (40 mg/140 cm^2^, Morus® tape L; Hisamitsu Pharmaceutical Co., Inc., Tosu, Japan), loxoprofen patch (100 mg/140 cm^2^, Loxonin^®^ tape; Daiichi‐Sankyo Co., Ltd., Tokyo, Japan), diclofenac patch (30 mg/140 cm^2^, Voltaren^®^ tape; Novartis Pharma, Tokyo, Japan), felbinac patch (70 mg/140 cm^2^, Seltouch^®^ pap; Pfizer Japan Inc., Tokyo, Japan), flurbiprofen patch (40 mg/140 cm^2^, Adofeed^®^ pap; Kaken Pharmaceutical Co., Ltd., Tokyo, Japan), and indomethacin patch (70 mg/140 cm^2^, Catlep^®^ pap; Teikoku Seiyaku Co., Ltd., Kagawa, Japan). These patches were purchased as commercially available products. Clinically, all of the drugs in this study were administered to the affected area in the form of a patch (10.0 × 14.0 cm).

### Human Recombinant COX‐1 and COX‐2 Assay

Diclofenac sodium salt, flurbiprofen, and indomethacin were purchased from Sigma‐Aldrich (St. Louis, MO) and felbinac was from Merck KGaA, (Darmstadt, Germany). Human COX‐2 recombinant protein was purchased from Cayman Chemical (Ann Arbor, MI). Recombinant COX‐1 was engineered as previously described [Sugimoto et al., 2016]. Enzyme activity was assayed in glass tubes each containing 200 μL of a reaction mixture consisting of 100 mM Tris–HCl buffer (pH 8.0), 1 μM hematin, 2 mM phenol, 12.6 μg/mL COX‐1, or 10.3 units/mL COX‐2, various concentrations of each NSAID (dissolved in dimethyl sulfoxide [DMSO]) or DMSO (control; final DMSO concentration: 1%)), and [1‐^14^C] arachidonic acid at 4.7 μmol/L (COX‐1) or 1.1 μmol/L (COX‐2). COX‐1 or COX‐2 enzymes were preincubated at 37**°**C for 15 min with drug solution or DMSO and the reaction started by adding [1‐^14^C] arachidonic acid. The reaction was stopped by adding 1 mL n‐hexane:ethyl acetate (2:1) after 2 min incubation. The aqueous phase was frozen, and the organic solvent phase discarded to remove any remaining [1‐^14^C] arachidonic acid. The extraction procedure was repeated three times. Since PGE_2_ selectively remained in the aqueous phase, the radioactivity of the aqueous phase was measured in a liquid scintillation counter. PG productions were calculated based on the conversion of [^14^C]‐arachidonic acid. The same experiments were repeatedly performed three or four times.

### Evaluation of Paw Hyperalgesia and Paw Swelling in AIA Rats

Seven‐week‐old male Lewis rats (Charles River Japan, Kanagawa, Japan) were used. All animal experiments were reviewed and approved by the Institutional Animal Care and Use Committee of Taisho Pharmaceutical Co., Ltd., and were in accordance with the Guidelines for Proper Conduct of Animal Experiments (Science Council of Japan, 2006). Arthritis was induced in Lewis rats by subcutaneously injecting adjuvant (0.8 mg of *Mycobacterium tuberculosis* H37 RA; DIFCO Laboratories, Detroit, MI) in 0.1 mL of liquid paraffin (Wako, Tokyo, Japan) into the left hind footpad (day 0). Twenty days after adjuvant injection, animals were divided into eight equal groups in such that the mean footpad volumes and the pain thresholds in all the groups were equivalent. Patches were applied to the right hind paw of the AIA‐treated rats for 6 h per day for 5 days. Doses were standardized by applying patches measuring 1.0 cm × 0.88 cm in size, based on the difference between human body weight and rat body weight. A group of rats to which no patches were applied was used as the control group. The measurement of pain thresholds was performed after each patch application for five days. The pain threshold was measured by counting the number of squeaking vocalizations induced by five consecutive gentle flexions of the ankle joint of the right paw. Footpad volume of the right hind paw was measured using a plethysmometer (Neuroscience, Tokyo, Japan) after 16 hours of the application of each patch for five days. The percent paw edema compared with that before patch treatment was calculated using the following formula:

Percent of paw edema (%)=(C−A)/(B−A)×100,

Where A is the paw volume before the injection of adjuvant solution, B is the paw volume before the patch application, and C is the paw volume after the patch administration. The area under the curve (AUC) of paw swelling and the paw hyperalgesia data were calculated using the area method from the time course data for the pain threshold and paw edema for the five days of patch application.

### Data Analysis

Results were expressed as the mean + or − SE. The differences between the control group and the experimental groups treated with patches and the differences between the SFPP group and the other patch groups were tested using a multiple comparison test, the Dunnett test, or the Steel test. A *P* value <0.05 was considered significant. For COX activity experiments, dose‐response curves for the inhibition percentage of PG production were fitted with a four‐parameter logistic function using a nonlinear least‐squares regression method. The IC_50_ values for COX inhibition activity were calculated as the concentration of the inhibitor that produced an inhibition level halfway between the maximum and minimum inhibition, fitted with a four‐parameter logistic function and calculated using a non‐linear least‐squares method.

## Results

### Inhibitory Effect on Human Recombinant COX‐1 or COX‐2 Activity

As shown in Table 1, SFP had the most potent inhibitory effect on both human COX‐1 and COX‐2 activities as compared to the NSAIDs, ketoprofen, loxoprofen, diclofenac, felbinac, flurbiprofen, and indomethacin under the same experimental conditions.

**Table 1:** Inhibitory Effect on Human Recombinant COX‐1 or COX‐2 Activity

Drug	COX‐1 IC_50_ (nmol/L) [95% CI]	COX‐2 IC_50_ (nmol/L) [95% CI]
S(+)‐flurbiprofen	8.97 [3.82–21.1]*	2.94 [1.41–6.12]*
Flurbiprofen	17.5 [7.37–41.4]	4.59 [2.68–7.85]
Ketoprofen	38.2 [6.87–213]*	26.1 [14.0–48.7]*
Loxoprofen‐SRS	1470 [1030–2100]*	25.9 [15.6–43.0]*
Diclofenac Na	13.0 [4.80–34.0]	4.0 [2.40–6.60]
Felbinac	14,000 [3,000–68,000]	12,000 [6,200–21,000]
Indomethacin	33.4 [9.24–121]	48.9 [41.4–57.9]

Data are expressed as the mean (95% confidence interval) obtained from four individual determinations or three individual determinations for diclofenac sodium salt (Na) and felbinac. IC_50_ values were estimated by non‐linear least‐squares method.

*Sugimoto et al. (2016).

### Effects of SFPP and Other NSAID Patches on Paw Hyperalgesia in AIA Rats with Repeated Applications

**Figure 1:**
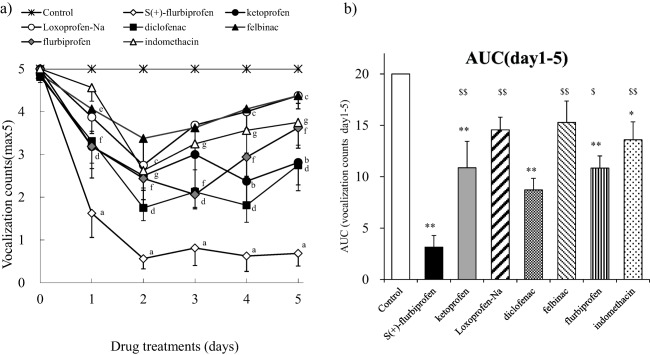
Analgesic effects on paws of AIA rats after multiple applications. On day 20 after the intraplantar injection of *M. tuberculosis* to induce arthritis, the drugs were applied to the right hind paw for 6 h per day for 5 days. Measurements of the pain response were performed on each day after the patch application using the method described in the “Materials and Methods” section. Each value and bar represents the mean ± SE of the results obtained in eight animals. (a) Time‐course of changes in paw hyperalgesia after the repeated application of each patch. a: SFPP, b: ketoprofen patch, c: loxoprofen patch, d; diclofenac patch, e; felbinac patch, f: flurbiprofen patch, and g: indomethacin patch. Differences relative to the value in the control group were compared using the Steel test, with *P* < 0.05 considered significant. (b) AUC (area under the curve) of changes in paw hyperalgesia from day 1 to day 5. **P < 0.01, significant difference relative to the value in the control group, $*P* < 0.05 and $$*P* < 0.01, significant difference relative to the value in the SFPP group (Dunnett test).

SFPP, diclofenac, and flurbiprofen exerted analgesic effects from the first to the last day of application compared with the control group (Fig. 1a). The other NSAIDs patch did not show a persistent analgesic effect lasting until the 5th day, but did show analgesic effects: days 4 and 5 for ketoprofen; days 2, 4, and 5 for loxoprofen; day 1 for felbinac; and days 2, 3, 4, and 5 for indomethacin. The inhibition rates on the 5th day for each patch were: SFPP 86%; ketoprofen 44%; loxoprofen 13%; diclofenac 45%, felbinac 13%, flurbiprofen 28%, and indomethacin 25%. The AUCs of the time‐course of the analgesic effect for the five days of application showed that SFPP and the diclofenac, ketoprofen, and flurbiprofen patches showed significant analgesic effects compared with the control group (Fig. 1b). The AUC analysis also indicated that SFPP showed a more potent analgesic effect than other NSAID patches, including ketoprofen, loxoprofen, felbinac, flurbiprofen, and indomethacin. The rank order of the AUC of the analgesic effect was SFPP > diclofenac, flurbiprofen, ketoprofen patches > indomethacin, loxoprofen, felbinac patches.

### Effects of Repeated Applications of SFPP and Other NSAID Patches on Paw Edema in AIA Rats

**Figure 2:**
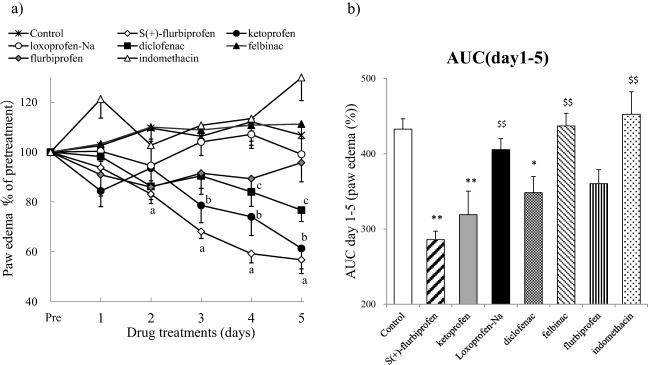
Anti‐inflammatory effects on paw edema of multiple applications in AIA rats. On day 20 after the intraplantar injection of *M. tuberculosis* to induce arthritis, the same size patch was applied to the right hind paw for 6 h per day for 5 days. Measurements of paw edema were performed on each day after the patch application using the method described in the “Materials and Methods” section. Each value and bar represents the mean + or − SE of the results obtained in eight animals. (a) Time‐course of changes in paw edema after the repeated application of each type of patch. a: SFPP, b: ketoprofen patch, c: loxoprofen patch, d: diclofenac patch, e: felbinac patch, f: flurbiprofen patch, and g: indomethacin patch. Differences relative to the value in the control group were compared using the Dunnett test, with *P* < 0.05 considered significant. (b) AUC (area under the curve) of changes in paw edema from day 1 to day 5. *P < 0.05 and **P < 0.01, significant difference relative to the value in the control group; $*P* < 0.05 and $$*P* < 0.01, significant difference relative to the value in the SFPP group (Dunnett test).

SFPP exerted an inhibitory effect on paw edema in a time‐dependent manner and decreased edema beginning on the second day after application, compared with the control group (Fig. 2a). The ketoprofen patch decreased edema beginning on the 3rd day after application and the diclofenac patch also decreased edema beginning on the 4th day after application, while the other NSAID patches (loxoprofen, felbinac, flurbiprofen, and indomethacin) failed to inhibit paw edema during the 5 days of application. The AUCs of the time‐course for the inhibitory effect on paw edema for the five days of application showed that SFPP and the diclofenac and ketoprofen patches exerted inhibitory effects versus the control group, and SFPP had a more potent inhibitory effect on edema than the NSAID patches including loxoprofen, felbinac and indomethacin (Fig. 2b).

## Discussion

In this study, we evaluated the analgesic effect of SFPP using a rat AIA model of pain. The inflammatory pain response to the joint flexion test in rat AIA models is valuable for assessing the effects of NSAIDs on chronic inflammatory pain, since the analgesic efficacy of NSAIDs in this model is correlated with the clinical therapeutic doses used for pain relief [Dubinsky et al., 1987]. In clinical settings, topical NSAID patches are applied for the alleviation of chronic inflammatory pain related to musculoskeletal disorders, including OA and RA, suggesting that an evaluation of the analgesic effects of multiple patch applications using the rat AIA model could provide a basis for predicting their clinical efficacy and be valuable for assessing the analgesic effects of NSAIDs. Previous studies reported the analgesic effects of topical NSAID patches using acute inflammatory pain models [Takayama et al., 2011; Komatsu et al., 2012] but the analgesic effect in a chronic inflammatory pain model using topical NSAID patches has not been previously reported. Previously, we reported that a single application of SFPP rapidly suppressed inflammatory pain in the AIA model, but the therapeutic efficacy of repeated SFPP applications remained to be elucidated in the rat AIA model. Conversely, with respect to edema, multiple applications of a ketoprofen patch had a more potent anti‐inflammatory effect than diclofenac patches in a rat AIA model. The results of our study showed that multiple applications of SFPP significantly improved pain and edema in a rat AIA model at the dose corresponding to the putative clinical dose range. In particular, SFPP showed a pronounced and rapid pain relief, and being effective from the first day of application with a plateau, and reached on the second day of application. Thus, our study suggests that SFPP might be a beneficial patch for clinical analgesic effect.

In inflammatory tissue, several mediators including histamine, bradykinin, nitric oxide, and the PGs are produced and contribute to the development of pain and inflammation. With respect to pain, PGE_2_ can excite nociceptors directly and potentiate the sensitizing effects of other pain mediators, including ATP, bradykinin, and capsaicin [Kawabata et al., 2011], inducing hyperalgesia at the inflammation tissue. Intraplantar injection of PGE_2_ in a carrageenan‐induced edema model can evoke prolonged tactile allodynia, including nociceptor hypersensitivity and a pain response [Amaya et al., 2006]. In addition, a neutralizing anti‐PGE_2_ monoclonal antibody prevented the development of hyperalgesia [Portanova et al., 1996].

Previously, we reported that a single application of SFPP rapidly decreased the PGE_2_ level in paw exudate and inflammatory pain in the AIA model suggesting that SFPP exerts a potent analgesic effect through the suppression of PGE_2_ production.

SFPP exerted an inhibitory effect on edema beginning on the second day of application, although it quickly exerted a potent analgesic effect beginning on the first day of application suggest results indicate that the analgesic effect preceded the suppression of edema. The other NSAIDs used in this study also produced an analgesic effect that preceded the suppression of edema. Edema is the result of plasmatic extravasation and increased blood flow in inflamed tissue that occurs via the PGE_2_‐mediated augmentation of arterial dilatation and increased microvascular permeability [Funk et al, 2001]. The present results suggested that the increase in volume attributable to edema could not be reduced until the extravasated plasma disappeared in the inflammatory region and that this might require several days to reduce the volume via the inhibition of PG production by NSAIDs.

In conclusion, multiple SFPP applications retained potent efficacy in providing inflammatory pain relief and anti‐inflammatory effects, suggesting that SFPP could be beneficial in the treatment of pain and inflammation in the clinical setting.

## Author Contributions

1. Conception and design of the study: M. Sugimoto, Y. Toda, S. Kaku, N. Otsuka
2. Acquisition of data, or analysis and interpretation of data: M. Sugimoto, Y. Toda, M. Hori, A. Mitani, T. Ichihara
3. Preparation of recombinant human COX‐1 protein: S. Sekine
4. Drafting of the article or critical revision for important intellectual content: M. Sugimoto, Y. Toda, S. Kaku, N. Otsuka, H. Matsumoto
5. Final approval of the version to be submitted: M. Sugimoto, Y. Toda, M. Hori, A. Mitani, T. Ichihara, S. Sekine, S. Kaku, N. Otsuka, H. Matsumoto

## Ethics of the Animal Experiments

All animal experiments reported here were reviewed and approved by the Institutional Animal Care and Use Committee of Taisho Pharmaceutical Co., Ltd., and were in accordance with the Guidelines for Proper Conduct of Animal Experiments (Science Council of Japan, 2006).
